# Traditional Herbal Medicine for Insomnia in Patients With Cancer: A Systematic Review and Meta-Analysis

**DOI:** 10.3389/fphar.2021.753140

**Published:** 2021-10-28

**Authors:** Jee-Hyun Yoon, Eun Hye Kim, Su Bin Park, Jee Young Lee, Seong Woo Yoon

**Affiliations:** ^1^ Korean Medicine Cancer Center, Kyung Hee University Hospital at Gangdong, Seoul, South Korea; ^2^ Jaseng Spine and Joint Research Institute, Jaseng Medical Foundation, Seoul, South Korea

**Keywords:** cancer, insomnia, traditional herbal medicine, meta-analyisis, systematic review

## Abstract

**Background:** Insomnia is one of the most prevalent cancer-related symptoms and has a severe impact on the quality of life. This study aimed to evaluate the efficacy and safety of traditional herbal medicine (THM) for improving sleep quality in patients with cancer.

**Methods:** Randomized controlled trials (RCTs) evaluating orally administered THM in a cancer population with insomnia were searched using nine electronic databases up to November 30, 2020. The outcome measurements were sleep quality measured by validated questionnaire such as the Pittsburgh Sleep Quality Index (PSQI), total effective rate, and adverse effects. The included studies were appraised using the Cochrane risk of bias tool and meta-analyzed. The quality of evidence was assessed using the Grading of Recommendations Assessment, Development, and Evaluation (GRADE) method.

**Results:** Fourteen RCTs were included in the systematic review, and 10 RCTs were analyzed quantitatively. Compared to hypnotics, THM showed a significant improvement in sleep quality by reducing the PSQI score [mean difference (MD) -2.25, 95% confidence interval (CI) −3.46 to −1.05, *I* ^*2*^ = 84%] and increasing the total effective rate [risk ratio (RR) 1.26, 95% CI 1.07 to 1.48, *I* ^*2*^ = 70%] with low quality of evidence. Compared to placebo, THM also reduced the PSQI score significantly (MD −2.56, 95% CI −3.81 to −1.31, *I* ^*2*^ = 91%) with moderate quality of evidence. The most frequently used herbs were *Ziziphus jujuba* Mill. No serious adverse events were observed.

**Conclusion:** This review suggests that THM may be an effective therapeutic option for insomnia in patients with cancer. However, considering the limited methodological qualities and inconsistent results of the included trials, further rigorous RCTs are required.

**Systematic Review Registration:** [https://www.crd.york.ac.uk/prospero], PROSPERO 2021 [CRD42021265070]

## Introduction

Insomnia is one of the most prominent complaints among patients with cancer (O’Donnell, 2004). It is defined as difficulty falling asleep, trouble staying asleep, early awakening, or nonrestorative sleep (Savard and Morin, 2001). The prevalence of insomnia in patients with cancer varies from 30 to 60%, which is considerably higher than that in the general population (O’Donnell, 2004; Induru and Walsh, 2014; Cleeland et al., 2013). In addition, many cancer patients do not appear to report symptoms of insomnia, assuming them to be a minor problem compared to a cancer diagnosis or treatment despite its prevalence and clinical significance, which results in insomnia remaining untreated (Induru and Walsh, 2014; Howell et al., 2014).

Insomnia and subsequent sleep disturbances can lead to fatigue, psychological disorders, and immunosuppression, which can significantly impair the quality of life and even affect the course of the disease (O’Donnell, 2004; Innominato et al., 2015). Non-pharmacological and pharmacological treatments are commonly used to treat insomnia. Cognitive behavioral therapy for insomnia (CBT-I) is recommended as a first treatment option for insomnia in the cancer population (Cleeland et al., 2013). Hypnotics are one of the most prescribed medications for patients with cancer (Stiefel et al., 1990). However, long-term adherence to CBT-I is difficult for patients with advanced cancer (Kvale and Shuster, 2006), and hypnotics have limited efficacy with adverse effects including dependence, impairment of memory or movement, and residual effects (Holbrook et al., 2000).

Traditional herbal medicine (THM) has been widely used to treat insomnia (Leach and Page, 2015). THM has been reported to modulate 5-hydroxytryptamine and gamma-aminobutyric acid (GABA) receptors (Cho et al., 2010), and affects brain enzymes associated with the GABA system (Awad et al., 2007). A previous systematic review and meta-analysis showed that THM had effects similar with hypnotics and a low frequency of adverse events compared to hypnotics in treating insomnia in the general population (Yeung et al., 2012).

In the cancer population, the causes of insomnia are multifactorial, and it may be due to cancer itself or a consequence of other cancer symptoms or related to cancer treatment (Induru and Walsh, 2014). Until now the clinical evidence of THM for managing insomnia in patients with cancer has not been established. This systematic review and meta-analysis of randomized controlled trials (RCTs) aimed to evaluate the efficacy and safety of THM in improving sleep quality in the cancer population.

## Methods

### Search Strategy

Two authors (J-HY and EK) independently conducted a systematic literature search in the following nine electronic databases: PubMed, Cochrane Library, Embase, China National Knowledge Infrastructure (CNKI), Japanese database (CiNii), and Korean databases (KMBASE, KISS, NDSL, OASIS). The databases were searched from inception to November 30, 2020. RCTs of THM for cancer patients with insomnia were included with no restrictions on publication date and language. The following keywords were used: neoplasm, cancer, insomnia, sleep disorders, herbal medicine, traditional Chinese medicine, traditional Korean medicine, and Kampo medicine. The details of our search strategies are presented in Supplementary Table S1. This systematic review and meta-analysis was conducted in accordance with the Preferred Reporting Items for Systematic Reviews and Meta-Analysis (PRISMA) guidelines (Moher et al., 2009), and the protocol of this review was registered in PROSPERO (registration no. CRD42021265070). Ethical approval is not required because all the research materials are published studies.

### Study Selection

Citations were independently evaluated by two investigators for eligibility at the title and abstract level and retrieved as full text if they were considered relevant. The inclusion criteria were as follows: 1) RCTs (parallel and/or crossover studies); 2) studies of cancer patients with insomnia; 3) studies with adult patients (age ≥18 years); 4) studies using oral administration of THM as an experimental intervention; 5) studies using conventional medicine, hypnotics, placebo, no treatment (waiting list group), usual care, or routine care as a control group; 6) studies reporting total effective rate or sleep quality measured with any grading scales, or validated questionnaires such as the Insomnia Severity Index (ISI) and the Pittsburgh Sleep Quality Index (PSQI). The total effective rate is one of the outcome measurements to assess clinical efficacy, the percentage of participants cured, markedly effective, and improved out of the total number enrolled. The exclusion criteria were as follows: 1) studies using intravenous administration or external preparation of THM; 2) studies reporting only quality of life; and 3) mixed interventions when oral administration of THM was not considered the main intervention. All instances of discordance were discussed between the investigators or, if necessary, arbitration by a third reviewer (SWY).

### Data Extraction

Two investigators independently abstracted data from the selected studies into a unified data statistics table. The following data were extracted: first author, publication year, sample size, patient characteristics (age, sex), type of cancer, diagnostic criteria of insomnia, intervention (composition, schedule, and duration), comparison, outcome measures, and adverse events. Outcomes were extracted at the longest duration of the complete follow-up. Any disagreement was resolved by consensus after discussion with a third investigator. If a study had missing information, we contacted the corresponding author when contact details were available.

### Quality Assessment

The methodological quality of included RCTs was assessed by two independent investigators using the Cochrane risk of bias tool with seven domains as follow: random sequence generation (selection bias), allocation concealment (selection bias), blinding of participants and personnel (performance bias), blinding of outcome assessment (detection bias), incomplete outcome data (attrition bias), selective reporting (reporting bias), and other bias (unclear distribution of prognostic factors). (Higgins et al., 2017). Each domain was appraised as “low-risk,” “high-risk,” or “uncertain risk.” Discrepancies were resolved by consulting a third investigator.

### Statistical Analysis

Data were pooled and analyzed using Review Manager (version 5.4). The mean difference (MD) for continuous variables and risk ratios (RRs) for dichotomous data with 95% confidence intervals (CIs) were used. If standard deviations were not reported, they were calculated from CIs or standard errors of the mean (Higgins et al., 2017). If the number of studies included in the comparison was more than four and had significant heterogeneity (>50%), a random-effects model was used; otherwise, a fixed-effects model was used (Tufanaru et al., 2015). Heterogeneity across studies was assessed by the Cochrane Chi-square test, with the significance threshold set at 0.10, and *I* ^*2*^ statistic, with a value of *I* ^*2*^ > 50% suggestive of significant heterogeneity (Higgins et al., 2003). Funnel plots were constructed to detect the potential publication bias if more than 10 studies were included in the meta-analysis. The Grading of Recommendations Assessment, Development, and Evaluation (GRADE) method was used to describe the quality of evidence for the results. It was classified as “high,” “moderate,” “low,” or “very low” for the outcome based on the risk of bias, inconsistency, indirectness, impression, and publication bias (Atkins et al., 2004).

## Results

### Study Selection

**FIGURE 1:**
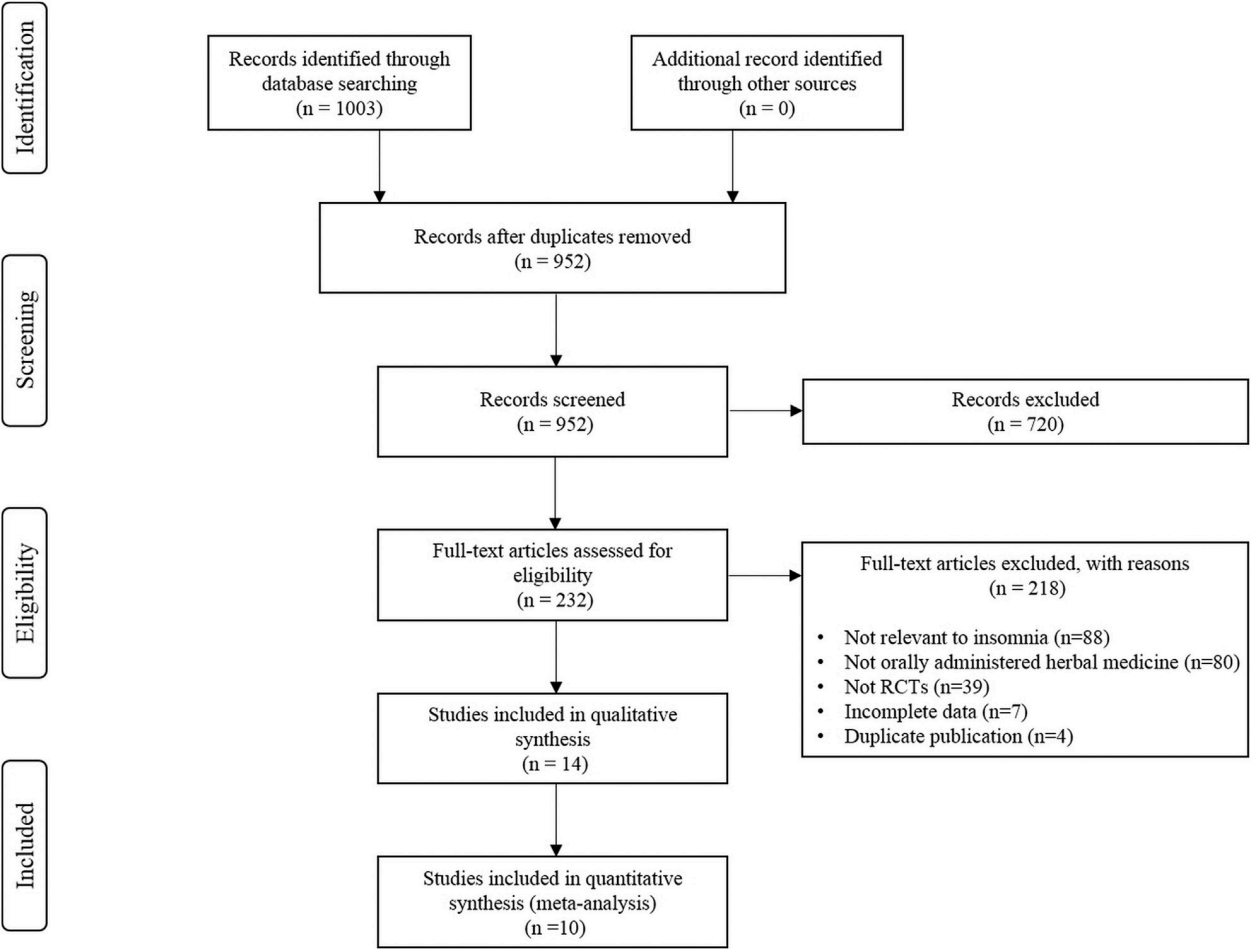
PRISMA flowchart of study selection.

The initial literature search yielded 1,003 studies, of which 51 were duplicate studies. Following the screening process, 720 studies were excluded based on the criteria used for screening the titles and abstracts. The remaining 232 studies were assessed for eligibility based on the full texts. Fourteen RCTs were finally included for the systematic review (Chen et al., 2009; Barton et al., 2011; Ji et al., 2016; Wang et al., 2016; Chen et al., 2017; Cheng et al., 2017; Gao et al., 2018; Lee et al., 2018; Liu, 2018; Cao, 2019; Hao, 2019; Moon et al., 2020; Pu et al., 2020; Wang et al., 2020), and data from ten studies were subjected to a meta-analysis (Ji et al., 2016; Wang et al., 2016; Chen et al., 2017; Cheng et al., 2017; Gao et al., 2018; Liu, 2018; Cao, 2019; Hao, 2019; Pu et al., 2020; Wang et al., 2020). A detailed flow chart of the literature search and the study selection is presented in Figure 1.

### Study Characteristics

The characteristics of the included RCTs are summarized in Table 1. Fourteen studies published between 2009 and 2020 were analyzed in this article (Chen et al., 2009; Barton et al., 2011; Ji et al., 2016; Wang et al., 2016; Chen et al., 2017; Cheng et al., 2017; Gao et al., 2018; Lee et al., 2018; Liu, 2018; Cao, 2019; Hao, 2019; Moon et al., 2020; Pu et al., 2020; Wang et al., 2020). Thirteen studies were carried out in Asia, including Korea and China (Chen et al., 2009; Ji et al., 2016; Wang et al., 2016; Chen et al., 2017; Cheng et al., 2017; Gao et al., 2018; Lee et al., 2018; Liu, 2018; Cao, 2019; Hao, 2019; Moon et al., 2020; Pu et al., 2020; Wang et al., 2020), and the remaining one study was carried out in the United States (Barton et al., 2011). The sample sizes in the studies varied from 22 to 202, with a total sample size of 1,020. Patients with various types of cancer, including lung and breast cancers, participated. The diagnosis of insomnia was based on the validated questionnaires with PSQI or ISI in five studies (Chen et al., 2009; Barton et al., 2011; Lee et al., 2018; Cao, 2019; Moon et al., 2020), and the Chinese Classification and Diagnostic Criteria of Mental Disorders third edition (CCMD 3) and International Classification of Sleep Disorders third edition (ICSD 3) in five studies (Ji et al., 2016; Wang et al., 2016; Cheng et al., 2017; Hao, 2019; Wang et al., 2020). Four studies did not report significant criteria (Chen et al., 2017; Gao et al., 2018; Liu, 2018; Pu et al., 2020).

**TABLE 1:** Basic characteristics of included studies.

References	Study design (n)	Mean age (I/C)	Cancer type	Diagnosis	Intervention group	Control group	Treatment duration	Outcome
Cao. (2019)	RCT (68)	45.11	Breast	PSQI >7	Chaihu Longgu Muli decoction	Estazolam	2 weeks	PSQI, TER
Chen et al., (2017)	RCT (60)	(56.7/60.5)	Lung	N/A	Guben Anshen decoction	Estazolam	4 weeks	PSQI, TER, TCMSS, KPS
Cheng et al. (2017)	RCT (90)	N/A	Lung	ICSD-3	Baihe Gujin Tang combined with Huanglain Ejiao Tang	Zolpidem	2 weeks	PSQI, TER, sleep quality
Gao et al. (2018)	RCT (60)	(56.12/57.11)	Lung	N/A	Guben Anshen decoction	Estazolam	4 weeks	PSQI, TER, TCMSS
Hao (2019)	RCT (60)	(59.07/55.14)	Lung, breast, gynecologic, others	CCMD-3	Chaihu Longgu Muli decoction	Estazolam	4 weeks	PSQI, TER
Ji et al. (2016)	RCT (80)	(55.23/54.16)	N/A	CCMD-3	Anshen Bukangling decoction	Estazolam	30 days	PSQI, TER
Liu (2018)	RCT (80)	(46.65/46.35)	N/A	N/A	Huanglian Wendan Longgu Muli decoction	Diazepam	30 days	Therapeutic efficiency, TER
Wang et al. (2016)	RCT (66)	N/A	N/A	CCMD-3	Kongsheng Zhenzhong pill	Estazolam	4 weeks	PSQI, TCM pattern Integral
Barton et al. (2011)	RCT (202)	(59.5/58.3)	Breast, colon, prostate, others	Sleeping difficulty ≥4	Valerian capsule	Placebo	8 weeks	PSQI, POMS, BFI
Pu et al. (2020)	RCT (92)	N/A	Lung, gynecologic, urologic, others	N/A	Suanzaoren tea	Placebo	10 days	PSQI, HDSS
Wang et al. (2020)	RCT (70)	N/A	Liver	CCMD-3	Suanzaoren decoction	Placebo	4 weeks	PSQI, TER, TCMSS, Lab data
Chen et al. (2009)	RCT (40)	(58.25/56.06)	N/A	PSQI >7	Warm settling decoction	Warm settling decoction + exercise	8 weeks	PSQI, BFI
Lee et al. (2018)	RCT (30)	(55.7/52.6)	Breast, lung, gastrointestinal, others	PSQI >5	Gamiguibi-tang	Usual care	2 weeks	ISI, BFI, BDI, MoCA
Moon et al. (2020)	RCT (22)	(63.0/63.0)	Lung, breast, liver, others	ISI ≥8	Cheonwangbosimdan	CBT-I	4 weeks	PSQI, ISI, ESS, SAS, BFI, EQ-5D

I, intervention; C, control; PSQI, pittsburgh sleep quality index; N/A, not available; ICSD-3, international classification of sleep Disorders 3rd edition; CCMD-3, Chinese classification and diagnostic criteria of mental disorders 3rd edition; ISI, insomnia severity index; CBT-I, cognitive behavioral therapy for insomnia; TER, total effective rate; TCMSS, traditional Chinese medicine syndrome scale; KPS, karnofsky performance score; POMS, profile of moods states; BFI, brief fatigue inventory; HDSS, hyperhidrosis disease severity scale; BDI, beck depression Inventory; MoCA, montreal cognitive assessment; ESS, epworth sleepiness scale; SAS, Zung self-rating anxiety scale; EQ-5D, EuroQol-5 dimensions.

The herbal formulas and the detailed single components of 14 RCTs included in the systematic review are presented in Supplementary Table S2 (Chen et al., 2009; Barton et al., 2011; Ji et al., 2016; Wang et al., 2016; Chen et al., 2017; Cheng et al., 2017; Gao et al., 2018; Lee et al., 2018; Liu, 2018; Cao, 2019; Hao, 2019; Moon et al., 2020; Pu et al., 2020; Wang et al., 2020). The most frequently used herbal formulae in 14 studies were Guben Anshen decoction (Chen et al., 2017; Gao et al., 2018) and Chaihu Longgu Muli decoction (Cao, 2019; Hao, 2019). The specific features of the single components used in 14 RCTs including the category of traditional usage, the frequency of herbs, the bioactive compounds, and the toxicity were demonstrated in Supplementary Table S3. The most frequently used herbs were *Ziziphus jujuba* Mill (*Z. jujuba*) (Ji et al., 2016; Chen et al., 2017; Gao et al., 2018; Lee et al., 2018; Moon et al., 2020; Pu et al., 2020; Wang et al., 2020) in nine studies and Fossilia Ossis Mastodi (Chen et al., 2009; Wang et al., 2016; Chen et al., 2017; Gao et al., 2018; Liu, 2018; Cao, 2019; Hao, 2019) was used in seven studies.

For comparison, eight studies used hypnotics including estazolam, zolpidem, and diazepam (Ji et al., 2016; Wang et al., 2016; Chen et al., 2017; Cheng et al., 2017; Gao et al., 2018; Liu, 2018; Cao, 2019; Hao, 2019), and three studies used placebo control (Barton et al., 2011; Pu et al., 2020; Wang et al., 2020). The other three studies used exercise with THM (Chen et al., 2009), usual care (Lee et al., 2018), and CBT-I (Moon et al., 2020). The duration of treatment ranged from 10 days to 8 weeks.

Most studies used the PSQI as a subjective outcome to assess the effect of THM (Chen et al., 2009; Barton et al., 2011; Ji et al., 2016; Wang et al., 2016; Chen et al., 2017; Cheng et al., 2017; Gao et al., 2018; Cao, 2019; Hao, 2019; Moon et al., 2020; Pu et al., 2020; Wang et al., 2020), and eight studies used the total effective rate (Ji et al., 2016; Chen et al., 2017; Cheng et al., 2017; Gao et al., 2018; Liu, 2018; Cao, 2019; Hao, 2019; Wang et al., 2020). Other outcomes included the ISI (Lee et al., 2018; Moon et al., 2020), Brief Fatigue Inventory (Chen et al., 2009; Barton et al., 2011; Lee et al., 2018; Moon et al., 2020), and Traditional Chinese Medicine Syndrome Scale (Chen et al., 2017; Gao et al., 2018; Wang et al., 2020).

### Risk of Bias in the Included Studies

**FIGURE 2:**
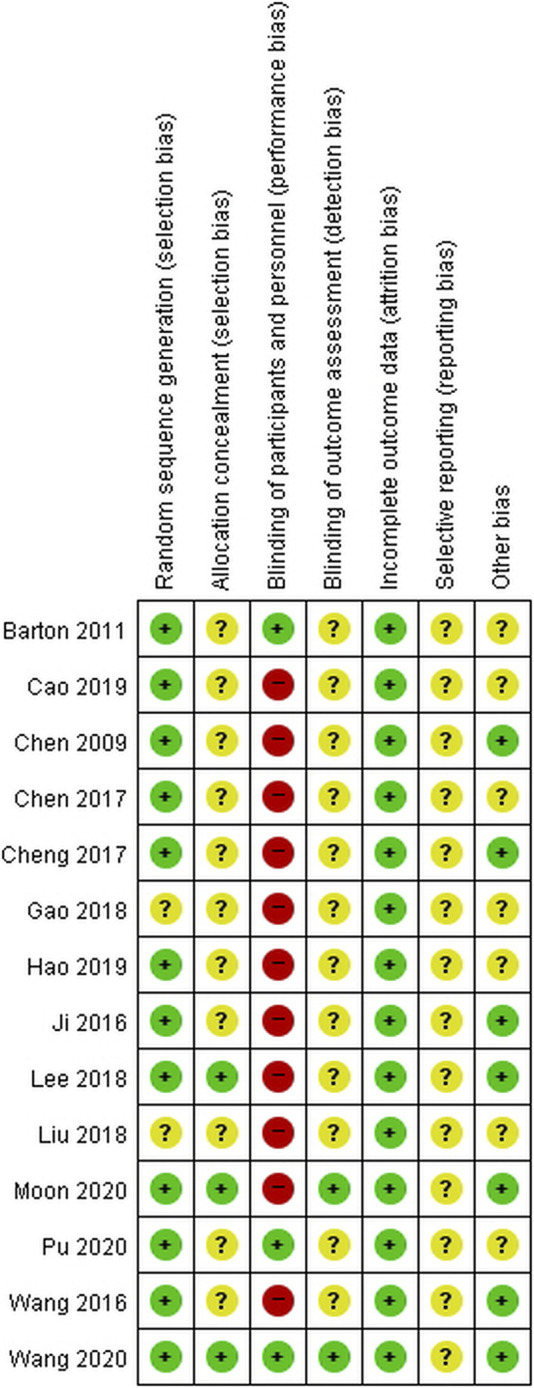
Risk of bias summary. +, low risk of bias; ?, unclear of bias; −, high risk of bias.

The risk of bias in the included studies is shown in Figure 2. Random sequence generation was adequately described in most of the studies but was unclear in two studies (Gao et al., 2018; Liu, 2018). In terms of allocation concealment, three studies reported the details of allocation procedure (Lee et al., 2018; Moon et al., 2020; Wang et al., 2020), whereas the remaining 11 studies had an unclear risk of bias (Chen et al., 2009; Barton et al., 2011; Ji et al., 2016; Wang et al., 2016; Chen et al., 2017; Cheng et al., 2017; Gao et al., 2018; Liu, 2018; Cao, 2019; Hao, 2019; Pu et al., 2020). Eleven studies did not blind either the participants or personnel or both (Chen et al., 2009; Ji et al., 2016; Wang et al., 2016; Chen et al., 2017; Cheng et al., 2017; Gao et al., 2018; Lee et al., 2018; Liu, 2018; Cao, 2019; Hao, 2019; Moon et al., 2020). However, three studies using placebo for the control group were evaluated as low in performance bias (Barton et al., 2011; Pu et al., 2020; Wang et al., 2020). Blinding of outcome investigators was presented in two studies and were assessed as low (Moon et al., 2020; Wang et al., 2020). However, other studies with no details were assessed as unclear (Chen et al., 2009; Barton et al., 2011; Ji et al., 2016; Wang et al., 2016; Chen et al., 2017; Cheng et al., 2017; Gao et al., 2018; Lee et al., 2018; Liu, 2018; Cao, 2019; Hao, 2019; Pu et al., 2020). All studies were evaluated as low in terms of incomplete outcome data and as uncertain in reporting bias (Chen et al., 2009; Barton et al., 2011; Ji et al., 2016; Wang et al., 2016; Chen et al., 2017; Cheng et al., 2017; Gao et al., 2018; Lee et al., 2018; Liu, 2018; Cao, 2019; Hao, 2019; Moon et al., 2020; Pu et al., 2020; Wang et al., 2020). Except for seven studies (Barton et al., 2011; Wang et al., 2016; Chen et al., 2017; Gao et al., 2018; Liu, 2018; Cao, 2019; Hao, 2019), the remaining studies were considered free of other biases with no difference at the baseline between the experiment and control groups (Chen et al., 2009; Ji et al., 2016; Cheng et al., 2017; Lee et al., 2018; Moon et al., 2020; Pu et al., 2020; Wang et al., 2020).

### Traditional Herbal Medicine Versus Hypnotics

Eight studies compared THM with hypnotics (Cao, 2019; Chen et al., 2017; Cheng et al., 2017; Gao et al., 2018; Hao, 2019; Ji et al., 2016; Liu, 2018; Wang et al., 2016), and the treatment duration of THM ranged from 14 to 30 days. Among the included studies, five studies had clear diagnostic criteria for insomnia (Cao, 2019; Cheng et al., 2017; Hao, 2019; Ji et al., 2016; Wang et al., 2016), whereas three studies did not (Chen et al., 2017; Gao et al., 2018; Liu, 2018). Three studies included lung cancer (Chen et al., 2017; Cheng et al., 2017; Gao et al., 2018), one study included breast cancer (Cao, 2019), one study included various cancer types (Hao, 2019), and the remaining three studies did not mention the type of cancer in the enrolled patients (Ji et al., 2016; Liu, 2018; Wang et al., 2016).

#### Pittsburgh Sleep Quality Index

**FIGURE 3:**
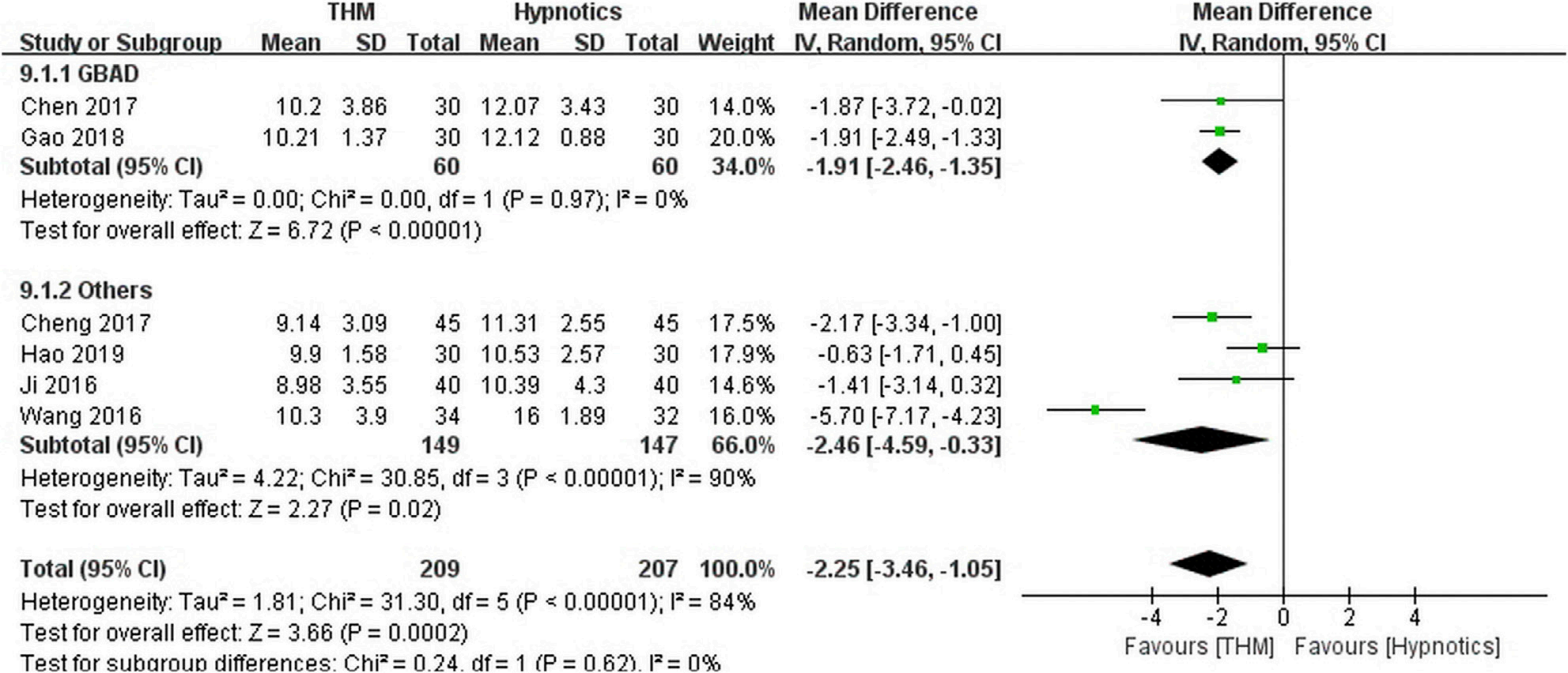
Forest plot of the pittsburgh sleep quality index for THM versus hypnotics. THM, traditional herbal medicine; GBAD, Guben Anshen decoction.

Six RCTs with a total of 416 patients provided adequate data on the PSQI score and were included in the meta-analysis (Figure 3) (Chen et al., 2017; Cheng et al., 2017; Gao et al., 2018; Hao, 2019; Ji et al., 2016; Liu, 2018; Wang et al., 2016). There was a statistically significant improvement in sleep quality with use of THM compared with the use of hypnotics (MD −2.25, 95% CI −3.46 to −1.05, *p* < 0.001), accompanied by a high grade of heterogeneity (*I* ^*2*^ = 84%). In the subgroup analysis, treatment with Guben Anshen decoction significantly improved sleep quality compared with hypnotics (MD −1.91, 95% CI −2.46 to −1.35, *p* < 0.001) (Chen et al., 2017; Gao et al., 2018). No evidence of heterogeneity was found (*I* ^*2*^ = 0%). The GRADE profile indicates that the quality of evidence was low for the PSQI outcome of THM compared to that of hypnotics, mainly due to methodological limitations and unexplained inconsistencies (Table 2).

**TABLE 2:** Summary of findings.

Traditional herbal medicine compared to hypnotics or placebo for insomnia in patients with cancer						
Patient or population: Patients with Cancer-related Insomnia						
Intervention: Traditional Herbal Medicine						
Comparison: Hypnotics						
Outcomes	Anticipated absolute effects* (95% CI)		Relative effect (95% CI)	No of participants (studies)	Certainty of the evidence (GRADE)	Comments
	Risk with Hypnotics	Risk with Traditional Herbal Medicine				
Pittsburgh Sleep Quality Index (PSQI)	The mean PSQI was 0	MD 2.25 lower (3.46 lower to 1.05 lower)	—	416 (6 RCTs)	⊕⊕○○	—
					LOW	
Total Effective Rate (TER)	631 per 1,000	794 per 1,000 (675–933)	RR 1.26 (1.07–1.48)	498 (7 RCTs)	⊕⊕○○	—
					LOW	
Patient or population: Patients with Cancer-related Insomnia						
Intervention: Traditional Herbal Medicine						
Comparison: Placebo						
Outcomes	Anticipated absolute effects* (95% CI)		Relative effect (95% CI)	No of participants (studies)	Certainty of the evidence (GRADE)	Comments
	Risk with Placebo	Risk with Traditional Herbal Medicine				
Pittsburgh Sleep Quality Index (PSQI)	The mean PSQI was 0	MD 2.56 lower (3.81 lower to 1.31 lower)	—	162 (2 RCTs)	⊕⊕⊕○	—
					MODERATE	
*The risk in the intervention group (and its 95% confidence interval) is based on the assumed risk in the comparison group and the relative effect of the intervention (and its 95% CI)						
CI: Confidence interval; MD: Mean difference; RR: Risk ratio						
GRADE Working Group grades of evidence						
High certainty: We are very confident that the true effect lies close to that of the estimate of the effect						
Moderate certainty: We are moderately confident in the effect estimate: The true effect is likely to be close to the estimate of the effect, but there is a possibility that it is substantially different						
Low certainty: Our confidence in the effect estimate is limited: The true effect may be substantially different from the estimate of the effect						
Very low certainty: We have very little confidence in the effect estimate: The true effect is likely to be substantially different from the estimate of effect						

#### Total Effective Rate

**FIGURE 4:**
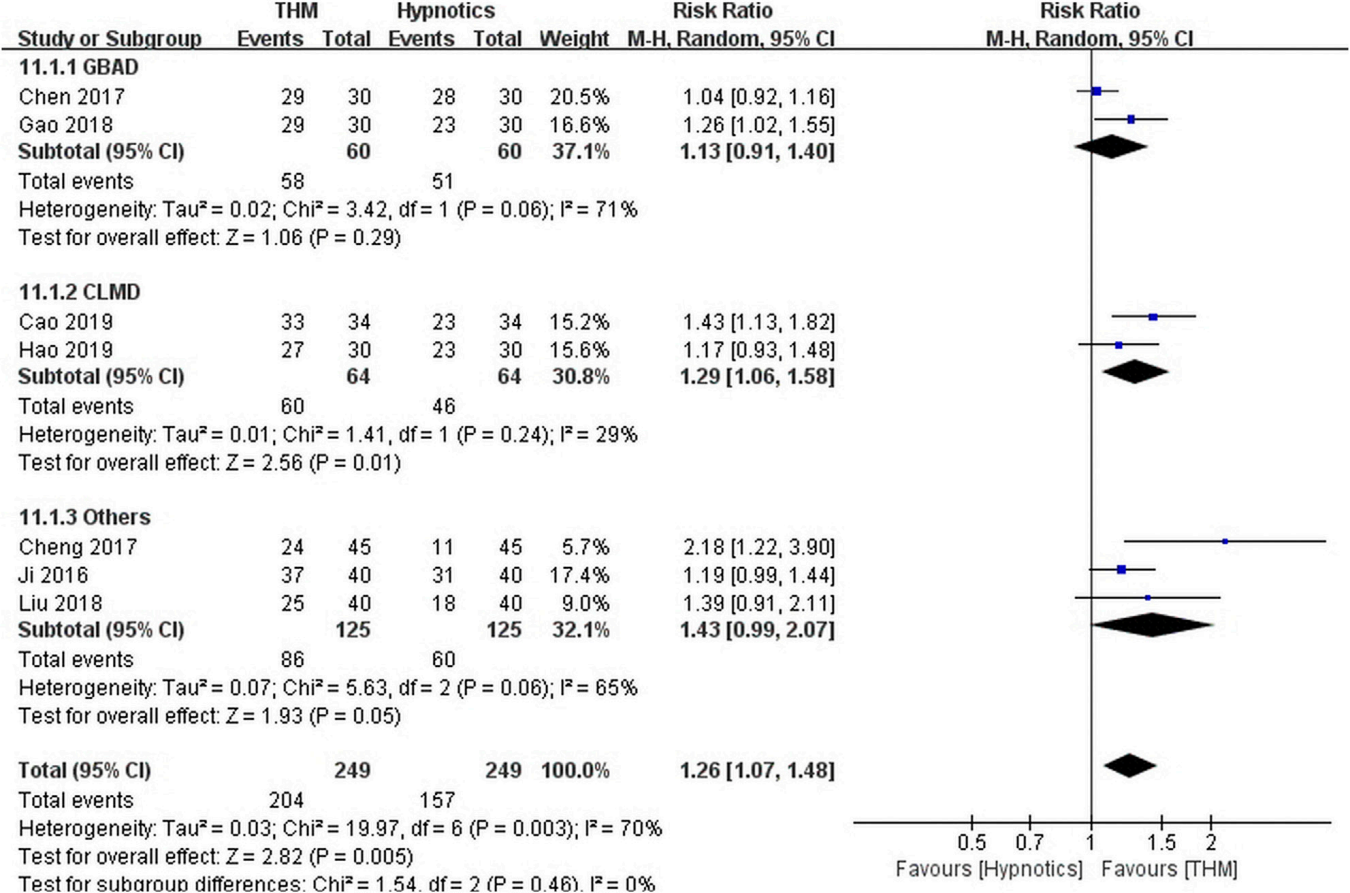
Forest plot of total effective rate for THM versus hypnotics. THM, traditional herbal medicine; GBAD, Guben Anshen decoction; CLMD, Chaihu Longgu Muli decoction.

Seven RCTs comprising 498 patients were included in the meta-analysis of the total effective rate (Figure 4) (Cao, 2019; Chen et al., 2017; Cheng et al., 2017; Gao et al., 2018; Hao, 2019; Ji et al., 2016; Liu, 2018). Overall, treatment with THM resulted in a statistically significant improvement compared to treatment with hypnotics (RR 1.26, 95% CI 1.07 to 1.48, *p* = 0.005), with high heterogeneity among the studies (*I* ^*2*^ = 70%). In the subgroup analysis, there was a statistically non-significant improvement in sleep quality with the use of Guben Anshen decoction compared to the use of hypnotics (RR 1.13, 95% CI 0.91 to 1.40, *p* = 0.29), along with high heterogeneity among the studies included (*I* ^*2*^ = 71%) (Chen et al., 2017; Gao et al., 2018). Treatment with Chaihu Longgu Muli decoction significantly improved sleep quality compared to treatment with hypnotics (RR 1.29, 95% CI 1.06 to 1.58, *p* = 0.01), with a low grade of heterogeneity (*I* ^*2*^ = 29%) (Cao, 2019; Hao, 2019). The GRADE profile indicated that the quality of evidence was low for the total effective rate outcome of THM compared to that of hypnotics, mainly due to methodological limitations and unexplained inconsistencies (Table 2).

### Traditional Herbal Medicine Versus Placebo

Three studies compared THM with placebo (Barton et al., 2011; Pu et al., 2020; Wang et al., 2020), and the treatment duration of THM ranged from 10 to 56 days. Among the included studies, two studies had clear diagnostic criteria for insomnia (Barton et al., 2011; Wang et al., 2020), whereas one study did not (Pu et al., 2020). Two studies included various cancer types (Barton et al., 2011; Pu et al., 2020), and the remaining one study included liver cancer (Wang et al., 2020).

#### Pittsburgh Sleep Quality Index

**FIGURE 5:**
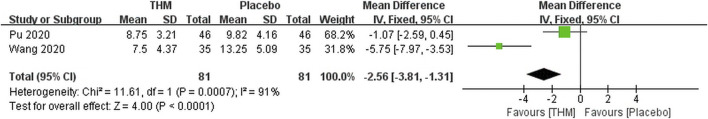
Forest plot of the pittsburgh sleep quality index of THM versus placebo. THM, traditional herbal medicine.

Two RCTs with a total of 162 patients reported adequate data on the PSQI score and were included in the meta-analysis (Figure 5) (Pu et al., 2020; Wang et al., 2020). There was a statistically significant improvement in sleep quality with the use of THM compared with that of placebo (MD -2.56, 95% CI -3.81 to -1.31, *p* < 0.001) accompanied by a high grade of heterogeneity (*I* ^*2*^ = 91%). Two studies included in the meta-analysis used *Z. jujuba* as the main herb. The GRADE profile indicates that the quality of evidence was moderate for the PSQI outcome of THM compared to that of placebo due to unexplained inconsistencies (Table 2).

### Traditional Herbal Medicine Versus Conventional Care

Three studies used conventional care as a control intervention (Chen et al., 2009; Lee et al., 2018; Moon et al., 2020). One study showed that THM, named Cheonwangbosimdan, had similar improvement in cancer-related insomnia, and better improvement in anxiety compared to CBT-I (Moon et al., 2020). Another study showed that THM, named Gamiguibi-tang, had significant improvement in cancer-related insomnia compared to usual care such as sleep hygiene education (Lee et al., 2018). The other study reported that THM with warm-settling decoction showed better improvement in cancer-related insomnia and fatigue when combined with exercise compared to THM treatment alone (Chen et al., 2009).

### Adverse Events

Adverse event monitoring was only reported in four RCTs (Barton et al., 2011; Chen et al., 2017; Cheng et al., 2017; Lee et al., 2018). No serious adverse events were observed. One study reported a significantly lower incidence rate of adverse events in the treatment group compared with the control group, but laboratory tests were not assessed through the study (Chen et al., 2017). Another study showed no significant difference between groups for the self-reported adverse events (i.e., headache, trouble waking, and nausea) (Barton et al., 2011). However, alkaline phosphatase elevation, assessed as grade 1 on the Common Terminology Criteria for Adverse Events (CTCAE) scale, had a significantly higher incidence in the placebo group (Barton et al., 2011), while the other two studies presented normal laboratory results including liver and renal function after THM administration (Cheng et al., 2017; Lee et al., 2018).

## Discussion

The present study reviewed 14 studies involving 1,020 patients with insomnia in patients with cancer, and 10 studies were included in the meta-analysis. The main finding of this study is that THM significantly improved insomnia in patients with cancer compared to hypnotics or placebo with low to moderate quality of evidence. In addition, Guben Anshen decoction, mainly composed of *Z. jujuba*, significantly reduced the PSQI score and Chaihu Longgu Muli decoction, mainly composed of Fossilia Ossis Mastodi, significantly improved the total effective rate in the subgroup analysis. Serious adverse effects have not been reported in a few studies assessing the safety of THM. However, it is not possible to define any superior effect of THM for insomnia in patients with cancer compared to hypnotics or placebo, given the small number of studies with low methodological quality.

Over 50% of patients with cancer experience insomnia and have overlooked it, assuming insomnia to be a normal and temporary reaction to cancer diagnosis or treatment (O’Donnell, 2004; Howell et al., 2014). According to guidelines for the treatment of insomnia in the general population, CBT-I is strongly recommended as the first-line treatment for chronic insomnia in adults with high-quality evidence. A pharmacological intervention can be offered if CBT-I is not sufficiently effective or unavailable. Hypnotics, including benzodiazepines, are effective in the short-term treatment of insomnia with weak recommendation and moderate quality of evidence. Complementary and alternative treatments, including acupuncture, meditative movement, aromatherapy, and homeopathy, are not recommended for insomnia treatment with weak recommendation and very low quality of evidence (Riemann et al., 2017).

To the best of our knowledge, there are no guidelines for insomnia in patients with cancer. The lack of standardized assessments and treatment guidelines for insomnia in patients with cancer makes management difficult (Induru and Walsh, 2014) and the implementation of CBT-I as a treatment model in cancer care requires further research, and their role in advanced disease may be limited (Kvale and Shuster, 2006; Pachman et al., 2012). Although hypnotics, including benzodiazepines and non-benzodiazepines, are often prescribed to cancer patients with insomnia, the effectiveness of these agents is not established (Paltiel et al., 2004). Moreover, there is a concern about drug-drug interactions given the polypharmacy inevitable in metastatic cancer (Induru and Walsh, 2014). Benzodiazepines with opioids are common with delirium, falls, and excess neuropsychological side effects. Non-benzodiazepines, which are better tolerated, may still cause light-headedness and somnolence (Savard and Morin, 2001; Induru and Walsh, 2014).

THM has been used extensively to treat insomnia in East Asian countries for thousands of years (Chen et al., 2011). Modern science has revealed that the anxiolytic and sedative properties of herbal insomnia medications involve targeting the GABAergic systems (Shi et al., 2014). The seed of *Z. jujuba* exerts sedative and hypnotic actions mediated primarily by the GABA system. Sanjoinine A, one of the major secondary metabolites of *Z. jujuba*, showed a similar effect with other GABA_A_ receptor agonists by activating glutamic acid decarboxylase and increasing GABA receptor γ-subunit expression (Ma et al., 2007). The other active compound in *Z. jujuba*, jujuboside A, modulates GABA_A_ receptor α- and β-subunit expression (Shergis et al., 2017). Fossilia Ossis Mastodi has been shown partial agonistic or subtype-selective modulation to GABA_A_ receptor (Ha et al., 2006). A prior systematic review demonstrated that in the general population, THM was equivalent to hypnotics in treating insomnia and had a lower frequency of adverse events compared to hypnotics, while Gui Pi Tang (Guibi-tang) was the most commonly used herbal formula and *Z. jujuba* was the most frequently used single herb (Yeung et al., 2012). A population-based pharmaco-epidemiologic study showed that diverse Chinese herbal medicine for insomnia in Taiwan were prescribed, while the commonly used herbal formula was Suanzaoren decoction with *Z. jujuba* as the main herb (Chen et al., 2011). A previous meta-analysis found that Zao Ren An Shen, the herbal formula containing the seed of *Z. jujuba*, had a superior effect on sleep quality to placebo and a similar effect to benzodiazepine receptor agonists (Birling et al., 2020). These prior findings of THM for insomnia in the general population are consistent with the results of our study in cancer patients including the major herbs and herbal formulas which were commonly used.

This review is the first attempt to focus on the hypnotic effect of THM in treating insomnia in patients with cancer and has the strength to follow the rigorous review process of Cochrane methodology, reporting standards such as PRISMA, and addressing quality of evidence using the GRADE system. Despite our rigorous attempts to identify all current evidence, this study has some limitations. First, significant heterogeneity was observed in the meta-analysis. The reason for the heterogeneity could be related to the use of various types of hypnotics and THM. Second, trials without double-blinding have an unavoidable risk of bias and methodological limitations of component trials may affect the finding of this review. Third, the long-term effect of THM on cancer-related insomnia could not be determined because the duration of THM treatment was short. Finally, since most of the studies were conducted in Asian countries, the investigated population may not represent a broad spectrum of cancer patients. Future RCTs are needed to provide higher-quality evidence for THM in treating insomnia in patients with cancer.

In conclusion, low to moderate quality evidence suggests that THM can improve insomnia in patients with cancer. To further understand the potential role of traditional herbal medicine in the treatment of cancer-related insomnia, more well-designed, double-blind, large-scale randomized controlled trials are needed. As options for the experimental intervention, herbal formulas mainly composed of *Z. jujuba* (i.e., Gamiguibi-tang, Cheonwangbosimdan) could be promising since *Z. jujuba* is the most frequently used herb in this review and prior studies. It is also valuable to evaluate the long-term efficacy and additional strategies for combining THM and conventional therapy to overcome the limitations of current treatment for insomnia in patients with cancer.

## Data Availability

The original contributions presented in the study are included in the article/Supplementary Material, further inquiries can be directed to the corresponding author.
