# Supplementary material for: Traditional Herbal Medicine for Insomnia in Patients With Cancer: A Systematic Review and Meta-Analysis
Source: Front Pharmacol. 2021 Oct 28;12:753140. doi: 10.3389/fphar.2021.753140 (PMC8581246; doi:10.3389/fphar.2021.753140)
Supplement: Supplementary file 1 [file Table1.DOCX]

**Supplementary Material S1.** Search terms for each database.

1. PubMed

#20 #3 and #11 and #19

#19 #12 or #13 or # 14 or # 15 or #16 or #17 or #18

#18 ('traditional Korean medicine' or 'traditional Chinese medicine' or 'traditional oriental medicine' or 'Kampo medicine' or herb* or decoction* or botanic*)

#17 "Medicine, Chinese Traditional"[MeSH]

#16 "Medicine, Korean Traditional"[MeSH]

#15 "Medicine, Kampo"[MeSH]

#14 "Herbal Medicine"[MeSH]

#13 "Plants, Medicinal"[MeSH]

#12 "Drugs, Chinese Herbal"[MeSH]

#11 #4 or #5 or #6 or #7 or #8 or #9 or #10

#10 (insomnia* or dyssomnia* or sleep* or wakeful*)

#9 "Wakefulness"[Mesh]

#8 "Sleep"[Mesh]

#7 "Sleep Initiation and Maintenance Disorders"[Mesh]

#6 "Sleep Disorders, Intrinsic"[Mesh]

#5 "Dyssomnias"[Mesh]

#4 "Sleep Wake Disorders"[Mesh]

#3 #1 or #2

#2 (neoplasm* or cancer* or carcino* or malignan* or tumor* or tumour*)

#1 "Neoplasms"[Mesh]

2. Cochrane Library

#1 MeSH descriptor: [Neoplasms] explode all trees

#2 neoplasm* or cancer* or carcino* or malignan* or tumor* or tumour*

#3 #1 OR #2

#4 MeSH descriptor: [Sleep Wake Disorders] explode all trees

#5 MeSH descriptor: [Dyssomnias] explode all trees

#6 MeSH descriptor: [Sleep Disorders, Intrinsic] explode all trees

#7 MeSH descriptor: [Sleep Initiation and Maintenance Disorders] explode all trees

#8 MeSH descriptor: [Sleep] explode all trees

#9 MeSH descriptor: [Wakefulness] explode all trees

#10 insomnia* or dyssomnia* or sleep* or wakeful*

#11 #4 OR #5 OR #6 OR #7 OR #8 OR #9 OR # 10

#12 MeSH descriptor: [Medicine, Kampo] explode all trees

#13 MeSH descriptor: [Medicine, Korean Traditional] explode all trees

#14 MeSH descriptor: [Medicine, Chinese Traditional] explode all trees

#15 #12 OR #13 OR #14

#16 #3 AND #11 AND #15

3. Embase

#16 #3 AND #11 AND #15

#15 #12 OR #13 OR #14

#14 'Kampo medicine'/exp

#13 'Korean medicine'/exp

#12 'Chinese medicine'/exp

#11 #4 OR #5 OR #6 OR #7 OR #8 OR #9 OR #10

#10 (insomnia* OR dyssomnia* OR sleep* OR wakeful*)

#9 'wakefulness'/exp

#8 'sleep'/exp

#7 'sleep initiation and maintenance disorders'/exp

#6 'sleep disorders, intrinsic'/exp

#5 'dyssomnias'/exp

#4 'sleep wake disorders'/exp

#3 #1 OR #2

#2 (neoplasm* or cancer* or carcino* or malignan* or tumor* or tumour*)

#1 'neoplasms'/exp

4. CNKI

#1 癌

#2 肿瘤

#3 cancer

#4 OR / 1-3

#5 睡眠障碍

#6 睡眠失调

#7 失眠症

#8 失眠

#9 睡眠质量

#10 不寐

#11 sleep disorders

#12 insomnia

#13 sleep quality

#14 OR / 5-13

#15 中药

#16 中医

#17 汤

#18 饮

#19 散

#20 汤剂

#21 丸

#22 中成药

#23 方剂

#24 中西医结合

#25 颗粒

#26 胶囊

#27 口服液

#28 Capsule

#29 Powder

#30 Herbal medicine

#31 Traditional Chinese medicine

#32 TCM

#33 Traditional medicine

#34 Decoction

#35 Chinese medicine

#36 OR / 15-35

#37 #4 AND #14 AND #36

5. KMBASE

#1 ([KEYWORD=cancer] OR [KEYWORD=neoplasms])

#2 ([ABSTRACT=insomnia] OR [ABSTRACT=sleep disorders])

#3 (([ABSTRACT=traditional medicine] OR [ABSTRACT=Korean medicine]) OR [ABSTRACT=herb])

#4 AND / 1-3

6. KISS

#1 cancer AND insomnia AND Korean medicine

#2 cancer AND insomnia AND traditional medicine

#3 OR / 1-2

7. NDSL

#1 ABSTRACT=cancer

#2 ABSTRACT=insomnia | sleep disorders

#3 ABSTRACT=Korean medicine | traditional medicine | herb

#4 AND / 1-3

#5 ABSTRACT=randomized

#6 AND / 4-5

8. OASIS

#1 cancer AND insomnia AND Korean medicine

#2 cancer AND insomnia AND traditional medicine

#3 cancer AND insomnia AND herb

#4 OR / 1-3

9. CiNii

#1 (Cancer OR neoplasms) AND (insomnia OR sleep disorders) AND (herb OR Korean medicine OR Chinese medicine OR Kampo medicine OR herbal medicine
