# Supplementary material for: Traditional Herbal Medicine for Insomnia in Patients With Cancer: A Systematic Review and Meta-Analysis
Source: Front Pharmacol. 2021 Oct 28;12:753140. doi: 10.3389/fphar.2021.753140 (PMC8581246; doi:10.3389/fphar.2021.753140)
Supplement: Supplementary file 2 [file Table2.docx]

**Supplementary Material S2.** Herbal formulas and its components in the included studies.

| **Study** | **Formulation** | **Source** | **Species, concentration** | **Quality control**  **reported?**  **(Y/N)** | **Chemical analysis**  **reported?**  **(Y/N)** |
| --- | --- | --- | --- | --- | --- |
| Cao. (2019) | Chaihu Longgu Muli Decoction | Beijing Puxiang Traditional Chinese Medicine Cancer Hospital | Bulb of *Fritillaria thunbergii* Miq., 20g  Herb of *Scutellaria barbata* D.Don, 20g  Shell of *Ostrea gigas* Thunberg, 20g  Fossilia Ossis Mastodi, 20g  Spike of *Prunella vulgaris* L., 15g  Rhizome of *Curcuma phaeocaulis* Valeton, 15g  Root of *Scutellaria baicalensis* Gerogi, 15g  Root of *Pseudostellaria heterophylla* (Miq.) Pax, 15g  Root and rhizome of *Glycyrrhiza uralensis* Fisch. ex DC., 10g  Rhizome of *Pinellia ternata* (Thunb.) Makino, 10g  Root of *Bupleurum chinense* DC., 10g | Y – Prepared according to Pharmacopoeia of the People's Republic of China | N |
| Chen et al. (2017) | Guben Anshen Decoction | Guangdong Second Traditional Chinese Medicine Hospital | Seed of *Ziziphus jujuba* Mill., 30g  Rhizome of *Dioscorea oppositifolia* L., 30g  Bulb of *Lilium lancifolium* Thunb., 30g  Aril of *Dimocarpus longan* Lour., 30g  Bark of *Eucommia ulmoides* Oliv., 30g  Root of *Nanhaia speciosa* (Champ. ex Benth.) J.Compton & Schrire, 30g  Fossilia Ossis Mastodi, 30g  Shell of *Ostrea gigas* Thunberg, 30g  Herb of *Eclipta prostrata* (L.) L., 20g  Herb of *Lophatherum gracile* Brongn., 10g  Plumule of *Nelumbo nucifera* Gaertn., 6g | Y – Prepared according to Pharmacopoeia of the People's Republic of China | N |
| Cheng et al. (2017) | Baihe Gujin Tang combined with Huanglain Ejiao Tang | Beijing Navy General Hospital | Root of *Ophiopogon japonicus* (Thunb.) Ker Gawl., 30g  Bulb of *Lilium lancifolium* Thunb., 15g  Prepared root of *Rehmannia glutinosa* (Gaertn.) DC., 15g  Root of *Rehmannia glutinosa* (Gaertn.) DC., 15g  Root of *Angelica sinensis* (Oliv.) Diels, 15g  Root of *Scrophularia ningpoensis* Hemsl., 15g  Bulb of *Fritillaria cirrhosa* D.Don, 15g  Root of *Paeonia lactiflora* Pall., 12g  Root and rhizome of *Glycyrrhiza uralensis* Fisch. ex DC., 10g  Root of *Platycodon grandiflorus* (Jacq.) A.DC., 10g  Gelatin obtained from the skin of *Equus qsinus* L., 10g  Root of *Scutellaria baicalensis* Gerogi, 9g  Rhizome of *Coptis chinensis* Franch., 6g | Y – Prepared according to Pharmacopoeia of the People's Republic of China | N |
| Gao et al. (2018) | Guben Anshen Decoction | Hangzhou First People's Hospital | Seed of *Ziziphus jujuba* Mill., 30g  Rhizome of *Dioscorea oppositifolia* L., 30g  Bulb of *Lilium lancifolium* Thunb., 30g  Aril of *Dimocarpus longan* Lour., 30g  Bark of *Eucommia ulmoides* Oliv., 30g  Root of *Nanhaia speciosa* (Champ. ex Benth.) J.Compton & Schrire, 30g  Fossilia Ossis Mastodi, 30g  Shell of *Ostrea gigas* Thunberg, 30g  Herb of *Eclipta prostrata* (L.) L., 20g  Herb of *Lophatherum gracile* Brongn., 10g  Plumule of *Nelumbo nucifera* Gaertn., 6g | Y – Prepared according to Pharmacopoeia of the People's Republic of China | N |
| Hao. (2019) | Chaihu Longgu Muli Decoction | Huian Hospital of Integrated Traditional Chinese and Western Medicine | Root of *Bupleurum chinense* DC.  Rhizome of *Pinellia ternata* (Thunb.) Makino  Sclerotium of *Poria cocos* (Schw.) Wolf  Twig of *Neolitsea cassia* (L.) Kosterm.  Root of *Scutellaria baicalensis* Gerogi  Root and rhizome of *Panax ginseng* C.A.Mey.  Fruit of *Ziziphus jujuba* Mill.  Fossilia Ossis Mastodi  Shell of *Ostrea gigas* Thunberg  Rhizome of *Zingiber officinale* Roscoe  Root and rhizome of *Rheum officinale* Baill. | Y – Prepared according to Pharmacopoeia of the People's Republic of China | N |
| Ji et al. (2016) | Anshen Bukangling Decoction | Tumor Hospital Affiliated to Nantong University | Root of *Codonopsis pilosula* (Franch.) Nannf. 15g  Root of *Astragalus mongholicus* Bunge, 15g  Prepared root of *Rehmannia glutinosa* (Gaertn.) DC., 15g  Sclerotium of *Poria cocos* (Schw.) Wolf, 12g  Seed of *Ziziphus jujuba* Mill., 12g  Root of *Angelica sinensis* (Oliv.) Diels, 10g  Rhizome of *Conioselinum anthriscoides* 'Chuanxiong', 10g  Root of *Paeonia lactiflora* Pall., 10g  Root of *Polygala tenuifolia* Willd., 10g  Fruit of *Schisandra chinensis* (Turcz.) Baill., 10g  Rhizome of *Anemarrhena asphodeloides* Bunge, 10g  Root and rhizome of *Glycyrrhiza uralensis* Fisch. ex DC., 6g | Y – Prepared according to Pharmacopoeia of the People's Republic of China | N |
| Liu. (2018) | Huanglian Wendan Longgu Muli Decoction | Laizhou Hospital of Traditional Chinese Medicine | Root of *Codonopsis pilosula* (Franch.) Nannf., 30g  Fossilia Ossis Mastodi, 30g  Shell of *Ostrea gigas* Thunberg, 30g  Sclerotium of *Poria cocos* (Schw.) Wolf, 20g  Root of *Bupleurum chinense* DC., 12g  Twig of *Neolitsea cassia* (L.) Kosterm., 9g  Rhizome of *Pinellia ternata* (Thunb.) Makino, 9g  Rhizome of *Zingiber officinale* Roscoe, 6g  Root of *Scutellaria baicalensis* Gerogi, 6g  Root and rhizome of *Rheum officinale* Baill., 6g | Y – Prepared according to Pharmacopoeia of the People's Republic of China | N |
| Wang et al. (2016) | Kongsheng Zhenzhong pill | Beijing Tcmages Pharmaceutical Co., Ltd. | Shell of *Trionyx sinensis* Wiegmann, 20g  Fossilia Ossis Mastodi, 15g  Rhizome of *Acorus calamus* var. *angustatus* Besser, 12g  Root of *Polygala tenuifolia* Willd., 10g | Y – Prepared according to Pharmacopoeia of the People's Republic of China | Y– HPLC |
| Barton et al. (2010) | Valerian capsule | Hi-Health | Root of *Valeriana officinalis* L. | Y – Prepared according to The United States Pharmacopeia–National Formulary | Y– HPLC |
| Pu et al. (2020) | Suanzaoren tea | Affiliated Drum Tower Hospital, Medical School of Nanjing University | Seed of *Ziziphus jujuba* Mill., 30g | Y – Prepared according to Pharmacopoeia of the People's Republic of China | N |
| Wang et al. (2020) | Suanzaoren Decoction | Tianjiang Pharmaceutical Co., Ltd. | Rhizome of *Conioselinum anthriscoides* 'Chuanxiong', 12g  Seed of *Ziziphus jujuba* Mill., 10g  Sclerotium of *Poria cocos* (Schw.) Wolf, 10g  Rhizome of *Anemarrhena asphodeloides* Bunge, 10g  Root and rhizome of *Glycyrrhiza uralensis* Fisch. ex DC., 6g | Y – Prepared according to Pharmacopoeia of the People's Republic of China | Y– HPLC |
| Chen et al. (2009) | Warm settling decoction | Yueyang Hospital Affiliated to Shanghai University of Traditional Chinese Medicine | Root of *Astragalus mongholicus* Bunge  Root of *Codonopsis pilosula* (Franch.) Nannf.  Prepared daughter root of *Aconitum carmichaelii* Debeaux  Fossilia Ossis Mastodi  Shell of *Ostrea gigas* Thunberg  Magnetite  Root of *Polygala tenuifolia* Willd.  Fruit of *Schisandra chinensis* (Turcz.) Baill.  Herb of *Epimedium brevicornu* Maxim. | Y – Prepared according to Pharmacopoeia of the People's Republic of China | N |
| Lee et al. (2018) | Gamiguibi-tang | Kracie Pharma, Ltd. | Root of *Panax ginseng* C.A.Mey., 3g  Root of *Bupleurum falcatum* L., 3g  Rhizome of *Atractylodes lancea* (Thunb.) DC., 3g  Sclerotium of *Poria cocos* (Schw.) Wolf, 3g  Seed of *Ziziphus jujuba* Mill., 3g  Aril of *Dimocarpus longan* Lour., 3g  Root of *Astragalus mongholicus* Bunge, 2g  Root of *Angelica acutiloba* (Siebold & Zucc.) Kitag., 2g  Fruit of *Gardenia jasminoides* J.Ellis, 2g  Root of *Polygala tenuifolia* Willd., 1.5g  Fruit of *Ziziphus jujuba* Mill., 1.5g  Root and rhizome of *Glycyrrhiza uralensis* Fisch. ex DC., 1g  Root of *Aucklandia costus* Falc., 1g  Rhizome of *Zingiber officinale* Roscoe, 0.5g | Y – Prepared according to The Japanese Pharmacopoeia | Y – HPLC |
| Moon et al. (2020) | Cheonwangbosimdan | Jung Woo Pharmaceutical Co., Ltd. | Root of *Panax ginseng* C.A.Mey., 6.25g  Sclerotium of *Poria cocos* (Schw.) Wolf, 6.25g  Root of *Platycodon grandiflorus* (Jacq.) A.DC., 6.25g  Root of *Polygala tenuifolia* Willd., 6.25g  Root of *Salvia miltiorrhiza* Bunge, 6.25g  Root of *Scrophularia buergeriana* Miq., 6.25g  Root of *Rehmannia glutinosa* (Gaertn.) DC., 5g  Rhizome of *Coptis chinensis* Franch., 2.5g  Root of *Angelica gigas* Nakai, 1.25g  Tuber of *Asparagus cochinchinensis* (Lour.) Merr., 1.25g  Fruit of *Schisandra chinensis* (Turcz.) Baill., 1.25g  Seed of *Platycladus orientalis* (L.) Franco, 1.25g  Seed of *Ziziphus jujuba* Mill., 1.25g | Y – Prepared according to The Korean Pharmacopoeia | Y – HPLC |
