# Supplementary material for: Traditional Herbal Medicine for Insomnia in Patients With Cancer: A Systematic Review and Meta-Analysis
Source: Front Pharmacol. 2021 Oct 28;12:753140. doi: 10.3389/fphar.2021.753140 (PMC8581246; doi:10.3389/fphar.2021.753140)
Supplement: Supplementary file 3 [file Table3.DOCX]

**Supplementary Material S3.** Characteristics of single components in the included studies.

| **Category of traditional usage** | **Frequency of herbs** | **Scientific name** | **Toxicity**  **(daily dose)** | **Bioactive compounds** | **References** |
| --- | --- | --- | --- | --- | --- |
| Tranquilizing | 9 | *Ziziphus jujuba* Mill. | Non-toxic | Sanjoinine A, jujubosides | Shergis et al. (2017) |
|  | 7 | Fossilia Ossis Mastodi | Non-toxic | Calcium carbonate, calcium phosphate | Zhang et al. (2011) |
|  | 6 | *Ostrea gigas* Thunberg | N/A | Calcium carbonate | Zhang et al. (2011) |
|  | 6 | *Poria cocos* (Schw.) Wolf | Non-toxic | Pachymic acid, poricoic acid B | Ríos. (2011) |
|  | 5 | *Polygala tenuifolia* Willd. | Non-toxic | Tenuifoliside B | Ikeya et al. (2004) |
|  | 3 | *Dimocarpus longan* Lour. | Non-toxic | Corilagin, ellagic acid | Yang et al. (2011) |
|  | 3 | *Lilium lancifolium* Thunb. | Non-toxic | Kaempferol, *p*-coumaric acid | Jin et al. (2012) |
|  | 2 | *Nelumbo nucifera* Gaertn | Non-toxic | Isoliensinine, neferine | Mukherjee et al. (2009) |
|  | 1 | *Acorus calamus* var. *angustatus* Besser | Mild toxic (3–10 g) | α-Asarone, β-asarone | Han et al. (2013) |
|  | 1 | Magnetite | Non-toxic | Iron oxide mineral magnetite | Wang et al. (1997) |
|  | 1 | *Platycladus orientalis* (L.) Franco | Non-toxic | d-Limonene, cedrol | Zhu et al. (2020) |
|  | 1 | *Valeriana officinalis* L. | Non-toxic | Valepotriates, linarin | Fernández et al. (2004) |
| Qi tonifying | 5 | *Glycyrrhiza uralensis* Fisch. ex DC. | Non-toxic | Glycyrrhetinic acid, liquiritin | Chen et al. (2014) |
|  | 3 | *Panax ginseng* C.A.Mey. | Non-toxic | Ginsenosides, ployacetylene | Choi. (2008) |
|  | 3 | *Astragalus mongholicus* Bunge | Non-toxic | APS, *Astragalus* saponins VII | Fu et al. (2014) |
|  | 3 | *Codonopsis pilosula* (Franch.) Nannf. | Non-toxic | Lobetyolin, lobetyolinin | Bailly et al. (2021) |
|  | 3 | *Schisandra chinensis* (Turcz.) Baill. | Non-toxic | Schisandrin, deoxyschisandrin | Sowndhararajan et al. (2018) |
|  | 2 | *Dioscorea oppositifolia* L. | Non-toxic | YP-1 | Zao et al. (2005) |
|  | 1 | *Pseudostellaria heterophylla* (Miq.) Pax | Non-toxic | Heterophyllin B, rhamnogalacturonan I | Hu et al. (2019) |
| Heat clearing | 4 | *Scutellaria baicalensis* Gerogi | Non-toxic | Baicalein, baicalin | Liao et al. (2021) |
|  | 2 | *Anemarrhena asphodeloides* Bunge | Non-toxic | Timosaponin BIII, trans-hinokiresinol | Wang et al. (2018) |
|  | 2 | *Coptis chinensis* Franch. | Mild toxic (2–5 g) | Berberine, coptisine | Wang J. et al. (2018) |
|  | 2 | *Lophatherum gracile* Brongn. | Non-toxic | Isoorientin, swertiajaponin | Tang et al. (2015) |
|  | 2 | *Rheum officinale* Baill. | Non-toxic | Emodin, aloe-emodin | Huang et al. (2007) |
|  | 1 | *Fritillaria thunbergii* Miq. | Non-toxic | Peimine, peiminine | Nile et al. (2021) |
|  | 1 | *Fritillaria cirrhosa* D.Don | Non-toxic | Imperialine, peiminine | Chen T. et al. (2020) |
|  | 1 | *Gardenia jasminoides* J.Ellis | Non-toxic | Geniposide, genipin | Chen L. et al. (2020) |
|  | 1 | *Prunella vulgaris* L. | Non-toxic | Ursolic acid, 2α-hydroxyursolic acid | Ryu et al. (2000) |
|  | 1 | *Scrophularia buergeriana* Miq. | Non-toxic | Buergeriside C_1_, buergeriside A_1_ | Shin et al. (2018) |
|  | 1 | *Scrophularia ningpoensis* Hemsl. | Non-toxic | Scrophuside, ningposides | Hua et al. (2014) |
|  | 1 | *Scutellaria barbata* D.Don | Non-toxic | Pheophorbide a | Tang et al. (2006) |
| Yin-blood tonifying | 4 | *Rehmannia glutinosa* (Gaertn.) DC. | Non-toxic | Catalpol, RPS | Zhang et al. (2008) |
|  | 2 | *Angelica sinensis* (Oliv.) Diels | Mild toxic (6–12 g) | Ferulic acid, Z-ligustilide | Chao et al. (2011) |
|  | 2 | *Eclipta prostrata* (L.) L. | Non-toxic | Echinocystic acid, eclalbasaponin II | Lee et al. (2008) |
|  | 2 | *Paeonia lactiflora* Pall. | Mild toxic (6–15 g) | Paeoniflorin | He et al. (2011) |
|  | 1 | *Angelica acutiloba* (Siebold & Zucc.) Kitag. | Non-toxic | Butylidene phthalide, furfural | Sowndhararajan et al. (2017)  Yun et al., 2017 |
|  | 1 | *Asparagus cochinchinensis* (Lour.) Merr. | Non-toxic | Quercetin, asparacoside | Son et al. (2013) |
|  | 1 | *Equus qsinus* | N/A | Collagen α1, collagen α2 | Wang et al. (2014) |
|  | 1 | *Ophiopogon japonicus* (Thunb.) Ker Gawl. | Non-toxic | Ophiopogonin D, saponins | Chen et al. (2016) |
|  | 1 | *Trionyx sinensis* Wiegmann | N/A | CTEP | Tang et al. (2013) |
| Exterior-releasing | 3 | *Bupleurum chinense* DC. | Mild toxic (3–10 g) | Saikosaponins A, saikosaponins D | Liu et al. (2017) |
|  | 3 | *Zingiber officinale* Roscoe | Non-toxic | 6-Gingerol | de Lima et al. (2108) |
|  | 1 | *Bupleurum falcatum* L. | Mild toxic (3–10 g) | Saikosaponin A | Park et al. (2002) |
| Dampness-phlegm resolving | 3 | *Pinellia ternata* (Thunb.) Makino, 10g | Average toxic (internal use, 3–9 g after processed) | Pinellic acid | Nagai et al. (2002) |
|  | 2 | *Platycodon grandiflorus* (Jacq.) A.DC. | Non-toxic | Platycodin D, PGS | Zhang et al. (2015) |
|  | 1 | *Atractylodes lancea* (Thunb.) DC. | Non-toxic | Atractylodin, atractylodinol | Nakai et al. (2003) |
| Blood activating | 2 | *Conioselinum anthriscoides* 'Chuanxiong' | Mild toxic (3–10 g) | Ligustilide, senkyunolide A | Ran et al. (2001) |
|  | 1 | *Curcuma phaeocaulis* Valeton | Non-toxic | Phaeocaulisin A, phaeocaulisin B | Liu et al. (2013) |
|  | 1 | *Salvia miltiorrhiza* Bunge | Non-toxic | Danshensu, salvianolic acid B | Wang et al. (2017) |
| Yang-tonifying | 2 | *Eucommia ulmoides* Oliv. | Non-toxic | Eucommiol, astragalin | Wang C.Y. et al. (2019) |
|  | 1 | *Aconitum carmichaelii* Debeaux | Average toxic (3–15 g after processed) | Aconitine, mesaconitine | Zhao et al. (2020) |
|  | 1 | *Epimedium brevicornu* Maxim. | Mild toxic (6–10 g) | Icariin, epimedin B | Meng et al. (2004) |
| Wind-dampness dispelling | 2 | *Nanhaia speciosa* (Champ. ex Benth.) J.Compton & Schrire | Non-toxic | Formononetin, maackiain | Zhao et al. (2017) |
| Qi-regulating | 1 | *Aucklandia costus* Falc. | Non-toxic | Costunolide, dehydrocostuslactone | Lin et al. (2015) |

APS, *Astragalus mongholicus* polysaccharides; CTEP, *Carapax Trionycis* extract peptide; N/A, not available; PGS, *Platycodon grandiflorus* saponins; RPS, *Rehmannia glutinosa* polysaccharides.

The toxicity of single components in the included trials was cited form Liu et al. (2020). The study provided non-toxic traditional Chinese medicines (TCMs) with potential toxicity and toxic-TCMs, documented in the Chinese Pharmacopeia, with the toxic grade (highly toxic, average toxic, and mild toxic) and daily dose for body weight 70 kg.

**References**

Bailly, C. (2021). Anticancer Properties of Lobetyolin, an Essential Component of Radix Codonopsis (Dangshen). *Nat Prod Bioprospect.* 11, 143–153. doi: 10.1007/s13659-020-00283-9

Chao, W. W., & Lin, B. F. (2011). Bioactivities of major constituents isolated from Angelica sinensis (Danggui). *Chin Med*. 6, 29. doi:10.1186/1749-8546-6-29

Chen, H., Zhang, X., Feng, Y., Rui, W., Shi, Z., and Wu, L. (2014). Bioactive components of *Glycyrrhiza uralensis* mediate drug functions and properties through regulation of CYP450 enzymes. *Mol Med Rep.* 10, 1355–1362. doi: 10.3892/mmr.2014.2331

Chen, L., Li, M., Yang, Z., Tao, W., Wang, P., Tian, X., et al. (2020). *Gardenia jasminoides* Ellis: Ethnopharmacology, phytochemistry, and pharmacological and industrial applications of an important traditional Chinese medicine. *J Ethnopharmacol*. 257, 112829. doi: 10.1016/j.jep.2020.112829

Chen, M. H., Chen, X. J., Wang, M., Lin, L. G., and Wang, Y. T. (2016). *Ophiopogon japonicus*—A phytochemical, ethnomedicinal and pharmacological review. *J Ethnopharmacol*. 181, 193–213. doi:10. 1016/j.jep.2016.01.037

Chen, T., Zhong, F., Yao, C., Chen, J., Xiang, Y., Dong, J., et al. (2020). A Systematic Review on Traditional Uses, Sources, Phytochemistry, Pharmacology, Pharmacokinetics, and Toxicity of Fritillariae Cirrhosae Bulbus. *Evid Based Complement Alternat Med*. 2020, 1536534. doi: 10.1155/2020/1536534

Choi K. T. (2008). Botanical characteristics, pharmacological effects and medicinal components of Korean *Panax ginseng* C A Meyer. *Acta Pharmacol Sin*. 29, 1109–1118. doi: 10.1111/j.1745-7254.2008.00869.x

de Lima, R., Dos Reis, A. C., de Menezes, A., Santos, J., Filho, J., Ferreira, J., et al. (2018). Protective and therapeutic potential of ginger (*Zingiber officinale*) extract and [6]-gingerol in cancer: A comprehensive review. *Phytother Res*. 32, 1885–1907. doi: 10.1002/ptr.6134

Fernández, S., Wasowski, C., Paladini, A. C., and Marder, M. (2004). Sedative and sleep-enhancing properties of linarin, a flavonoid-isolated from *Valeriana officinalis*. *Pharmacol Biochem Behav*. 77, 399–404. doi: 10.1016/j.pbb.2003.12.003

Fu, J., Wang, Z., Huang, L., Zheng, S., Wang, D., Chen, S., et al. (2014). Review of the botanical characteristics, phytochemistry, and pharmacology of *Astragalus membranaceus* (Huangqi). *Phytother Res.* 28, 1275–1283. doi: 10.1002/ptr.5188

Han, P., Han, T., Peng, W., and Wang, X. R. (2013). Antidepressant-like effects of essential oil and asarone, a major essential oil component from the rhizome of *Acorus tatarinowii*. *Pharm Biol*. 51, 589–594. doi: 10.3109/13880209.2012.751616

He, D. Y., and Dai, S. M. (2011). Anti-inflammatory and immunomodulatory effects of *Paeonia lactiflora* Pall., a traditional Chinese herbal medicine. *Front Pharmacol*. 2, 10. doi: 10.3389/fphar.2011.00010

Hu, D. J., Shakerian, F., Zhao, J., and Li, S. P. (2019). Chemistry, pharmacology and analysis of *Pseudostellaria heterophylla*: a mini-review. *Chin Med.* 14, 21. doi: 10.1186/s13020-019-0243-z

Hua, J., Qi, J., and Yu, B. Y. (2014). Iridoid and phenylpropanoid glycosides from *Scrophularia ningpoensis* Hemsl. and their α-glucosidase inhibitory activities. *Fitoterapia*. 93, 67–73. doi: 10.1016/j.fitote.2013.11.011

Huang, Q., Lu, G., Shen, H. M., Chung, M. C., and Ong, C. N. (2007). Anti-cancer properties of anthraquinones from rhubarb. *Med Res Rev*. 27, 609–630. doi:10.1002/med.20094

Ikeya, Y., Takeda, S., Tunakawa, M., Karakida, H., Toda, K., Yamaguchi, T., et al. (2004). Cognitive improving and cerebral protective effects of acylated oligosaccharides in *Polygala tenuifolia*. *Biol Pharm Bull*. 27, 1081–1085. doi: 10.1248/bpb.27.1081

Jin, L., Zhang, Y., Yan, L., Guo, Y., and Niu, L. (2012). Phenolic compounds and antioxidant activity of bulb extracts of six Lilium species native to China. *Molecules*. 17, 9361–9378. doi: 10.3390/molecules17089361

Lee, M. K., Ha, N. R., Yang, H., Sung, S. H., Kim, G. H., and Kim, Y. C. (2008). Antiproliferative activity of triterpenoids from *Eclipta prostrata* on hepatic stellate cells. *Phytomedicine*. 15, 775–780. doi: 10.1016/j.phymed.2007.10.004

Liao, H., Ye, J., Gao, L., and Liu, Y. (2021). The main bioactive compounds of *Scutellaria baicalensis* Georgi. for alleviation of inflammatory cytokines: A comprehensive review. *Biomed Pharmacother*. 133, 110917. doi: 10.1016/j.biopha.2020.110917

Lin, X., Peng, Z., and Su, C. (2015). Potential anti-cancer activities and mechanisms of costunolide and dehydrocostuslactone. *Int J Mol Sci*. 16, 10888–10906. doi: 10.3390/ijms160510888

Liu, R., Li, X., Huang, N., Fan, M., and Sun, R. (2020). Toxicity of traditional Chinese medicine herbal and mineral products. *Adv Pharmacol*. 87, 301–346. doi: 10.1016/bs.apha.2019.08.001

Liu, X., Latkolik, S., Atanasov, A. G., Kunert, O., Pferschy-Wenzig, E. M., Heiss, E. H., et al. (2017). *Bupleurum chinense* Roots: a Bioactivity-Guided Approach toward Saponin-Type NF-κB Inhibitors. *Planta Med*. 83, 1242–1250. doi: 10.1055/s-0043-118226

Liu, Y., Ma, J., Zhao, Q., Liao, C., Ding, L., Chen, L., et al. (2013). Guaiane-type sesquiterpenes from *Curcuma phaeocaulis* and their inhibitory effects on nitric oxide production. *J Nat Prod*. 76, 1150–1156. doi: 10.1021/np400202f

Meng, F. H., Li, Y. B., Xiong, Z. L., Jiang, Z. M., and Li, F. M. (2005). Osteoblastic proliferative activity of *Epimedium brevicornum* Maxim. *Phytomedicine*. 12, 189–193. doi: 10.1016/j.phymed.2004.03.007

Mukherjee, P. K., Mukherjee, D., Maji, A. K., Rai, S., and Heinrich, M. (2009). The sacred lotus (*Nelumbo nucifera*) - phytochemical and therapeutic profile. *J Pharm Pharmacol*. 61, 407–422. doi: 10.1211/jpp/61.04.0001

Nagai, T., Kiyohara, H., Munakata, K., Shirahata, T., Sunazuka, T., Harigaya, Y., et al. (2002). Pinellic acid from the tuber of *Pinellia ternata* Breitenbach as an effective oral adjuvant for nasal influenza vaccine. *Int Immunopharmacol*. 2, 1183–1193. doi: 10.1016/s1567-5769(02)00086-3

Nakai, Y., Kido, T., Hashimoto, K., Kase, Y., Sakakibara, I., Higuchi, M., et al. (2003). Effect of the rhizomes of *Atractylodes lancea* and its constituents on the delay of gastric emptying. *J Ethnopharmacol*. 84, 51–55. doi: 10.1016/s0378-8741(02)00260-x

Nile, S. H., Su, J., Wu, D., Wang, L., Hu, J., Sieniawska, E., et al. (2021). *Fritillaria thunbergii* Miq. (Zhe Beimu): A review on its traditional uses, phytochemical profile and pharmacological properties. *Food Chem Toxicol*. 153, 112289. doi: 10.1016/j.fct.2021.112289

Oguri, K., Kawase, M., Harada, K., Shimada-Takaura, K., Takahashi, T., and Takahashi, K. (2016). Longgu (Fossilia Ossis Mastodi) alters the profiles of organic and inorganic components in Keishikaryukotsuboreito. *J Nat Med*. 70, 483–491. doi: 10.1007/s11418-015-0952-2

Park, K. H., Park, J., Koh, D., and Lim, Y. (2002). Effect of *saikosaponin-A*, a triterpenoid glycoside, isolated from *Bupleurum falcatum* on experimental allergic asthma. *Phytother Res*. 16, 359–363. doi: 10.1002/ptr.903

Ran, X., Ma, L., Peng, C., Zhang, H., and Qin, L. P. (2011). *Ligusticum chuanxiong* Hort: a review of chemistry and pharmacology. *Pharm Biol*. 49, 1180-1189. doi:10.3109/13880209.2011.576346

Ríos J. L. (2011). Chemical constituents and pharmacological properties of Poria cocos. *Planta Med*. 77, 681–691. doi: 10.1055/s-0030-1270823

Ryu, S. Y., Oak, M. H., Yoon, S. K., Cho, D. I., Yoo, G. S., Kim, T. S., et al. (2000). Anti-allergic and anti-inflammatory triterpenes from the herb of *Prunella vulgaris*. *Planta Med*. 66, 358–360. doi:10.1055/s-2000-8531

Shergis, J. L., Ni, X., Sarris, J., Zhang, A. L., Guo, X., Xue, C. C., et al. (2017). Ziziphus spinosa seeds for insomnia: A review of chemistry and psychopharmacology. *Phytomedicine*. 34, 38–43. doi: 10.1016/j.phymed.2017.07.004

Shin, H., Medriano, C. A., Park, B., Park, Y. H., and Lee, K. Y. (2018). Screening and identification of neuroprotective compounds from *Scrophularia buergeriana* using cell extraction coupled with LC-MS. *J Pharm Biomed Anal*. 148, 355–360. doi: 10.1016/j.jpba.2017.10.018

Son, H. L., and Anh, N. P. (2013). Phytochemical composition*, in vitro* antioxidant and anticancer activities of quercetin from methanol extract of *Asparagus cochinchinensis* (LOUR.) Merr. tuber. *J Med Plant Res*. 7, 3360–3366. doi: 10.5897/JMPR2013.5257

Sowndhararajan, K., Deepa, P., Kim, M., Park, S. J., and Kim, S. (2017). A Review of the Composition of the Essential Oils and Biological Activities of Angelica Species. *Sci Pharm*. 85, 33. doi: 10.3390/scipharm85030033

Sowndhararajan, K., Deepa, P., Kim, M., Park, S. J., and Kim, S. (2018). An overview of neuroprotective and cognitive enhancement properties of lignans from *Schisandra chinensis*. *Biomed Pharmacother*. 97, 958–968. doi: 10.1016/j.biopha.2017.10.145

Tang, P. M., Chan, J. Y., Au, S. W., Kong, S. K., Tsui, S. K., Waye, M. M., et al. (2006). Pheophorbide a, an active compound isolated from *Scutellaria barbata*, possesses photodynamic activities by inducing apoptosis in human hepatocellular carcinoma. *Cancer Biol Ther*. 5, 1111–1116. doi:10.4161/cbt.5.9.2950

Tang, Q., Shao, M., Wang, Y., Zhao, H., Fan, C., Huang, X., et al. (2015). Simultaneous Determination of 10 Bioactive Components of *Lophatherum gracile* Brongn by HPLC-DAD. *J Chromatogr Sci*. 53, 963–967. doi:10.1093/chromsci/bmu160

Tang, Y., Hu, C., and Liu, Y. (2013). Effect of bioactive peptide of *Carapax Trionycis* on TGF-β1-induced intracellular events in hepatic stellate cells. *J Ethnopharmacol*. 148, 69–73. doi: 10.1016/j.jep.2013.03.067

Wang, C. Y., Tang, L., He, J. W., Li, J., and Wang, Y. Z. (2019). Ethnobotany, Phytochemistry and Pharmacological Properties of *Eucommia ulmoides*: A Review. *Am J Chin Med*. 47, 259–300. doi: 10.1142/S0192415X19500137

Wang, D., Ru, W., Xu, Y., Zhang, J., He, X., Fan, G., Mao, B., Zhou, X., & Qin, Y. (2014). Chemical constituents and bioactivities of *Colla corii asini*. *Drug Discov Ther*. 8, 201–207. doi:10.5582/ddt.2014.01038

Wang, J., Wang, L., Lou, G. H., Zeng, H. R., Hu, J., Huang, Q. W., et al. (2019). Coptidis Rhizoma: a comprehensive review of its traditional uses, botany, phytochemistry, pharmacology and toxicology. *Pharm Biol*. 57, 193–225. doi: 10.1080/13880209.2019.1577466

Wang, L., Ma, R., Liu, C., Liu, H., Zhu, R., Guo, S., et al. (2017). *Salvia miltiorrhiza*: A Potential Red Light to the Development of Cardiovascular Diseases. *Curr Pharm Des*. 23, 1077–1097. doi: 10.2174/1381612822666161010105242

Wang, R., Huang, Y., Zhu, W., Zhang, H., and Sun, S. (1997). Pharmacological Study on Magnetite. *Zhongguo Zhong Yao Za Zhi*. 22, 305–309

Wang, Z., Cai, J., Fu, Q., Cheng, L., Wu, L., Zhang, W., et al. (2018). Anti-Inflammatory Activities of Compounds Isolated from the Rhizome of *Anemarrhena asphodeloides*. *Molecules.* 23, 2631. doi: 10.3390/molecules23102631

Yang, B., Jiang, Y., Shi, J., Chen, F., and Ashraf, M. (2011). Extraction and pharmacological properties of bioactive compounds from longan (*Dimocarpus longan* Lour.) fruit — A review. *Food Res Int*. 44, 1837–1842. doi: 10.1016/j.foodres.2010.10.019.

Yun, J. W., Kwon, E., Kim, S. H., You, J. R., Kim, Y. S., Park, I. A., et al. (2017). Preclinical safety assessment of *Angelica acutiloba* using a 13-week repeated dose oral toxicity study in rats. *Lab Anim Res*. 33, 223–230. doi: 10.5625/lar.2017.33.3.223

Zhang, H., Zhang, L., and Liu, Y. (2011). Studies on chemical components and pharmacological activities of Os Draconis (Longgu) and Ostreae Concha. *Zhongguo Zhong Yao Za Zhi*. 36, 1839–1840.

Zhang, L., Wang, Y., Yang, D., Zhang, C., Zhang, N., Li, M., et al. (2015). *Platycodon grandiflorus* - an ethnopharmacological, phytochemical and pharmacological review. *J Ethnopharmacol*. 164, 147–161. doi: 10.1016/j.jep.2015.01.052

Zhang, R. X., Li, M. X., and Jia, Z. P. (2008). *Rehmannia glutinosa*: review of botany, chemistry and pharmacology. *J Ethnopharmacol*. 117, 199–214. doi: 10.1016/j.jep.2008.02.018

Zhao, G., Kan, J., Li, Z., and Chen, Z. (2005). Structural features and immunological activity of a polysaccharide from *Dioscorea opposita* Thunb roots**.** *Carbohydr Polym*. 61, 125–131. doi: [10.1016/j.carbpol.2005.04.020](https://doi.org/10.1016/j.carbpol.2005.04.020)

Zhao, L., Sun, Z., Yang, L., Cui, R., Yang, W., and Li, B. (2020). Neuropharmacological effects of Aconiti Lateralis Radix Praeparata. *Clin Exp Pharmacol Physiol*. 47, 531–542. doi: 10.1111/1440-1681.13228

Zhao, Z., Liu, P., Ma, S., Wang, S., Li, A., Liu, J., et al. (2017). Botanical Characteristics, Chemical and Nutritional Composition and Pharmacological and Toxicological Effects of Medicinal and Edible Plant *Millettia speciosa* Champ. *Food Science*. 38, 293–306. doi: [10.7506/spkx1002-6630-201709046](https://doi.org/10.7506/spkx1002-6630-201709046)

Zhu, J. J., Yang, J. J., Wu, G. J., and Jiang, J. G. (2020) Comparative antioxidant, anticancer and antimicrobial activities of essential oils from *Semen Platycladi* by different extraction methods. *Ind Crops Prod*. 146, 112206. doi: 10.1016/j.indcrop.2020.112206
